# Sox11 Reduces Caspase-6 Cleavage and Activity

**DOI:** 10.1371/journal.pone.0141439

**Published:** 2015-10-27

**Authors:** Elaine Waldron-Roby, Janine Hoerauf, Nicolas Arbez, Shanshan Zhu, Kirsten Kulcsar, Christopher A. Ross

**Affiliations:** 1 Division of Neurobiology, Department of Psychiatry and Behavioral Sciences, Johns Hopkins University School of Medicine, CMSC 8-121, 600 North Wolfe Street, Baltimore, MD, 21287, United States of America; 2 Department of Neurology, Johns Hopkins University School of Medicine, CMSC 8-121, 600 North Wolfe Street, Baltimore, MD, 21287, United States of America; 3 Department of Pharmacology, Johns Hopkins University School of Medicine, CMSC 8-121, 600 North Wolfe Street, Baltimore, MD, 21287, United States of America; 4 Department of Neuroscience, Johns Hopkins University School of Medicine, CMSC 8-121, 600 North Wolfe Street, Baltimore, MD, 21287, United States of America; 5 Program in Cellular and Molecular Medicine, Johns Hopkins University School of Medicine, CMSC 8-121, 600 North Wolfe Street, Baltimore, MD, 21287, United States of America; McGill University, CANADA

## Abstract

The apoptotic cascade is an orchestrated event, whose final stages are mediated by effector caspases. Regulatory binding proteins have been identified for caspases such as caspase-3, -7, -8, and -9. Many of these proteins belong to the inhibitor of apoptosis (IAP) family. By contrast, caspase-6 is not believed to be influenced by IAPs, and little is known about its regulation. We therefore performed a yeast-two-hybrid screen using a constitutively inactive form of caspase-6 for bait in order to identify novel regulators of caspase-6 activity. Sox11 was identified as a potential caspase-6 interacting protein. Sox11 was capable of dramatically reducing caspase-6 activity, as well as preventing caspase-6 self- cleavage. Several regions, including amino acids 117–214 and 362–395 within sox11 as well as a nuclear localization signal (NLS) all contributed to the reduction in caspase-6 activity. Furthermore, sox11 was also capable of decreasing other effector caspase activity but not initiator caspases -8 and -9. The ability of sox11 to reduce effector caspase activity was also reflected in its capacity to reduce cell death following toxic insult. Interestingly, other sox proteins also had the ability to reduce caspase-6 activity but to a lesser extent than sox11.

## Introduction

Caspases are proteolytic enzymes critical for the orchestration of apoptosis, the final stages of which are mediated by effector caspases -3, -6, and -7 [1]. These effector caspases are activated via cleavage by other caspases [2]. However, it is becoming increasingly evident that certain effector caspases, including caspase-6, have more complex roles [3]. For example, unlike caspase-3 and 7, overexpression of caspase-6 in HEK cells does not result in apoptosis, suggesting differences in function and regulation [4,5]. Furthermore, caspase-6 and caspase-3 mediate degeneration of axons during normal development, which could potentially be involved in both neuronal cell death but also pruning of neuronal connections [6,7,8].

Caspase-6 has also been proposed to be involved in the cleavage of neurodegenerative disease-related proteins, such as huntingtin in Huntington’s disease (HD) [9]. Other proteins central to neurodegenerative diseases, such as APP, presenilin, and tau in AD are also substrates for caspase-6 [10,11,12,13,14,15,16,17]. Furthermore, caspase-6 activity seems to be increased in the diseases which it has substrates, e.g. HD and AD [18,19,20].

While few protein interaction partners for caspase-6 have been described, even less information concerning the regulation of caspase-6 activation is known [21,22]. Caspase-6 can activate itself upon over-expression [4]. Additionally, multiple caspases have been shown to be capable of activating caspase-6, these include caspase-1, caspase-9, caspase-3 and -7 [1,23,24]. Regulatory binding proteins have been identified for other caspases such as caspase-3, -7, -8, and -9 [25,26,27,28]. Many of these proteins belong to the inhibitor of apoptosis (IAP) family. For instance, c-IAP-1 and c-IAP-2 specifically inhibit caspase -3 and -7, respectively, but not other caspases [25,29]. By contrast, caspase-6 is not believed to be influenced by IAPs i.e. it does not interact with IAPs nor is it inhibited by IAPs.

In this study, we sought to identify novel caspase-6 interacting proteins which might be important in regulating caspase-6 activity. In an effort to identify proteins which bind preferentially to the zymogen, and thus might have regulatory roles, we used catalytically inactive caspase-6 as bait. The transcription factor, sox11 was identified as a candidate caspase-6 interactor. Sox11 is a member of the subgroup C family and has many interesting roles in development, cancer, nerve regeneration and adult neurogenesis [30,31,32,33]. Interestingly, sox11 has already been implicated in caspase-6 expression patterns. Knockdown of sox11 in the mantle cell lymphoma cell line, Grant 519 caused a significant reduction in caspase-6 RNA [34]. Another study also found caspase-6 RNA expression to be downregulated in primary limb buds isolated from sox4, sox11 conditional null allele mice [31]. Together these findings postulate a mediatory role for sox11 in caspase-6 expression. When co-expressed with caspase-6, sox11 was capable of dramatically blocking caspase-6 activity, and this ability was required the presence of an intact nuclear localization signal as well as several specific regions within the protein (i.e. amino acids 117–214 and its c-terminal domain). Sox11 was also capable of negatively impacting other effector caspases, but not initiator caspases -8 and -9. Finally, we report that sox11 overexpression can prevent cell death and other sox proteins may have the capacity to inhibit caspase-6 activity.

## Results

### Sox11 Is a Candidate Caspase-6 Binding Partner

In an effort to enrich for regulatory binding partners rather than substrates, we used a catalytically inactive caspase-6 as bait to screen a human fetal brain cDNA library. Yeast provides a suitable model system for studying caspase interactions, since yeast lack most of the caspases found in eukaryotic organisms, so exogenous caspase-6 is less likely to be cleaved and activated by other caspases. While over-expression of caspase-6 alone can induce autoactivation, the presence of a mutated catalytic site in full length caspase-6 prevents self-cleavage [4]. A total of 10 million clones were screened and resulted in over 100 positive colonies.

**Fig 1:**
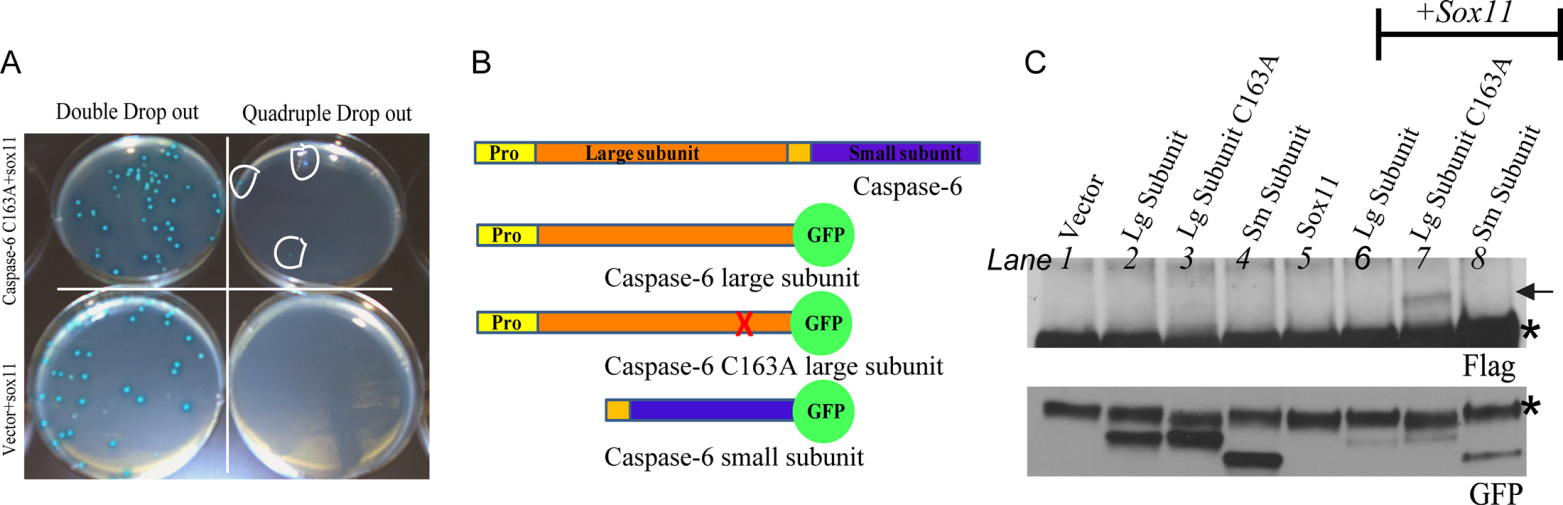
Confirmation of positive interactions in yeast and cells. (A) Bait (caspase-6 construct) and candidate prey (sox11 construct from library screen) were cotransformed into yeast and plated on selective media. Both bait and candidate prey plasmids can grow on double drop-out plates. Only colonies resulting from a genuine protein-protein interaction can grow on quadruple drop-out plates. Positive clones are circled in white. As expected, the negative control (vector+sox11) produced no colonies with quadruple drop-out. (B) Illustration of caspase-6 and the constructs used for co-immunoprecipitation. Yellow and orange bars denote propeptide. (C) Caspase-6 was immunoprecipitated from lysates using anti-GFP tagged caspase-6 subunits alone and with sox11. Lysates were immunoprecipitated using anti-GFP and probed with anti-flag. Top panel shows an interaction of the large caspase-6 subunit catalytically inactive with flag-tagged sox11 (Lane 7). The lower panel shows GFP tagged proteins were successfully imunoprecipitated. * indicates IgGs.

A collection of prey plasmids were rescued and positive interactions between prey and bait were confirmed in yeast. Only one was confirmed, and is shown in Fig 1a. This plasmid was sequenced and determined to encode a portion of the Sox11 protein. We next attempted to confirm the protein-protein interaction in cells. HEK 293FT cells were transiently transfected with sox11 and either procaspase-6 which auto-activates or a constitutively inactive form, where the catalytic site residue was mutated to an alanine (C163A). We did not detect a positive interaction between sox11 and procaspase-6 or caspase-6 C163A (data not shown). We repeated these interaction studies using caspase-6 large and small subunits (Fig 1b), and used a GFP tag on caspase-6 subunits in order to prolong their half-life. We confirmed the interaction of sox11 with the caspase-6 large subunit, containing the cysteine to alanine mutation (C163A) (Fig 1c, lane 7). Sox11 did not interact with the wildtype caspase-6 large subunit or small subunit (lanes 6 and 8, respectively).

### Sox11 Specifically Reduces Caspase-6 Activity

**Fig 2:**
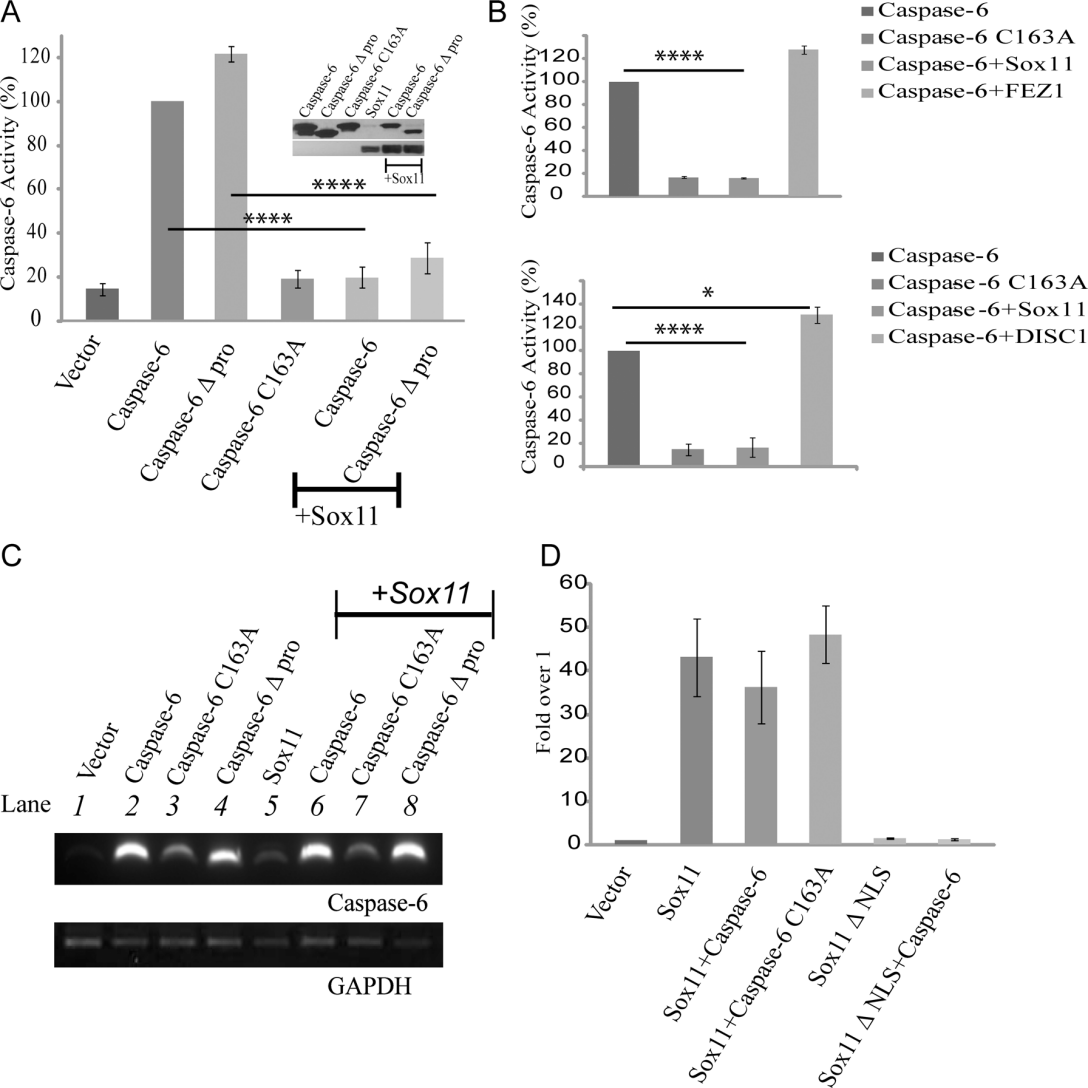
Sox11 reduces caspase-6 activity to control levels. (A) Lysates from HEK293FT cells were collected and assayed for caspase-6 activity via a luciferase based substrate assay system. NB caspase-6 Δpro refers to caspase-6 Δprodomain. The western blot shows caspase-6 activity is decreased in the presence of sox11. Caspase-6 activity was reduced to control levels by sox11. Statistical analysis showed this reduction was very significant (n = 3, one-way anova, **** p<0001). (B) Negative control experiments were performed using two other proteins, FEZ1 and DISC1. Neither FEZ1 nor DISC1 caused a significant decrease in caspase-6 activity but a one-way anova, followed by a Sidak’s multiple comparsion test showed DISC1 caused a slight increase in caspase-6 activity (n = 3, **** p<0001, *<0.05) (C) RNA was extracted from HEK293FT cells transfected for 24hours with indicated plasmids. Reverse transcription and PCR amplification using caspase-6 primers showed no significant change in mRNA levels between single and co-transfected samples (compare lanes 2, 3, and 4 with 6, 7, and 8, respectively). GAPDH levels were equal across all samples tested (n = 3). (D) A luciferase based reporter assay was used to assay sox11 transcriptional activity in the presence of caspase-6. Sox11 transactivation potential was not significantly affected by caspase-6 or caspase-6 C163A.

Having identified sox11 as a potential caspase-6 regulatory binding partner, we next wanted to determine if sox11 might affect caspase-6 activity. A luciferase based caspase-6 substrate assay was employed. This assay can measure the amount of active caspase-6 present in a given sample based on the amount of substrate cleaved. A clear rise in activity could be measured for both caspase-6 and caspase-6 with the prodomain already removed (Δ pro) but not caspase-6 C163A (Fig 2a). Therefore the assay is capable of detecting caspase-6 activity specifically. When either caspase-6 or caspase-6 Δ prodomain were co-transfected with sox11, caspase-6 activity levels were reduced to control levels (Fig 2a). Sox11 co-expression also resulted in a partial reduction of caspase-6 protein levels. In order to confirm the specificity of sox11 mediated reduction in caspase-6 activity, we co-transfected caspase-6 with two other proteins, FEZ1 which was identified from the yeast-two hybrid screen and DISC1 which is a known susceptibility protein for Schizophrenia. Using a caspase-6 substrate assay we were able to demonstrate that while sox11 completely blocked caspase-6 activity neither FEZ1 nor DISC1 had any effect (Fig 2b).

To assure that the sox11 mediated reduction of caspase-6 activity was not due to transcriptional repression, we performed RT-PCR analysis. The presence of sox11 did not affect caspase-6 mRNA levels compared to single transfections (Fig 2c, compare lanes 2, 3, and 4 with 6, 7, and 8, respectively). We also wanted to determine whether the ability of sox11 to inhibit caspase-6 impeded its transactivation potential. Sox11 transcriptional activity was assayed using a luciferase reporter plasmid (6FXO-p89Luc reporter) [35]. While sox11 was able to activate transcription, sox11 not containing a NLS (Δ NLS) was incapable of activating reporter activity (Fig 2d). Sox11 transactivation potential was not affected by cotransfection with caspase-6 (Fig 2d).

### Sox11 Prevents Caspase-6 Self-Cleavage

**Fig 3:**
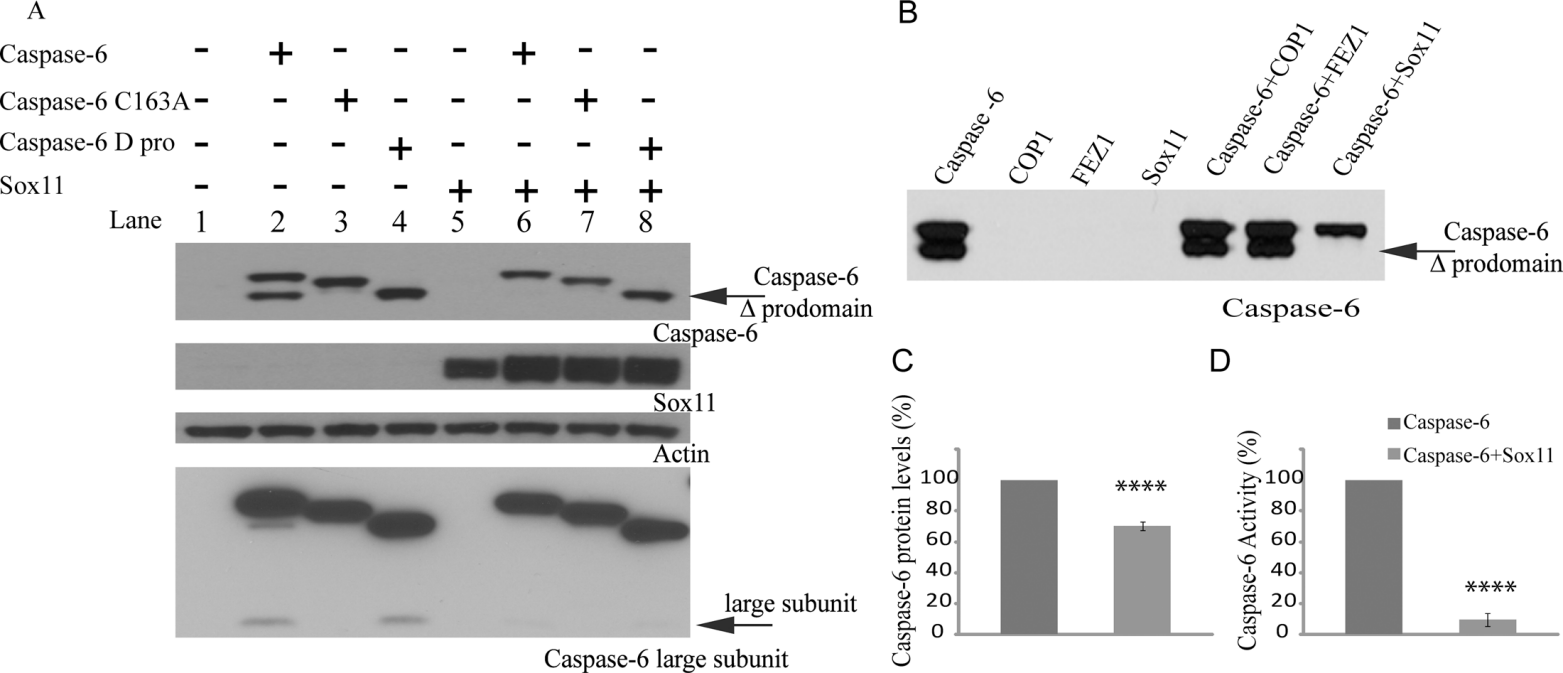
Sox11 prevents caspase-6 autocleavage. (A) Protein lysates were harvested and separated on a 4–12% bis-tris gel. Top panel shows a western blot of caspase-6 cleavage, the slower migrating band represents the uncleaved caspase-6 zymogen (note caspase-6 runs slighter higher than caspase-6 C163A because of an N-terminal flag tag) and the lower band represents caspase-6 following removal of its prodomain (lane 2). Cleavage of caspase-6 into caspase-6 Δprodomain is diminishedd in the presence of sox11 (lane 6). The lower panel is a western blot probed with an antibody capable of detecting the caspase-6 large subunit. Generation of the caspase-6 large subunit was suppressed in the presence of sox11 (compare lanes 2 and 4 with lanes 6 and 8, respectively). (B) Negative control experiments were performed using two other proteins identified in the yeast-two hybrid screen, COP1 and FEZ1. Neither FEZ1 nor COP1 affected caspase-6 self-cleavage. (C and D) Caspase-6 protein levels quantified using densitometry showed sox11 to significantly decrease caspase-6 protein levels by 30% but caspase-6 activity is completely blocked in the presence of sox11 (n = 8, unpaired t-test, **** p<0.0001).

Since caspase-6 activity depends on its cleavage, we performed western blot studies to determine whether caspase-6 was still undergoing cleavage in the presence of sox11 (Fig 3a). Previous publications have shown that overexpression of caspase-6 can result in its self-cleavage [4]. Transfection of caspase-6 alone resulted in its cleavage producing two distinct bands, the upper representing the full length protein and the lower representing caspase-6 post prodomain cleavage (Fig 3a, lane 2). In order to better resolve caspase-6 from caspase-6 after prodomain removal, caspase-6 was flag tagged and thus runs slightly higher than caspase-6 C163A. Unlike caspase-6 wildtype, caspase-6 C163A was not cleaved despite containing small and large subunit cleavage sites, which implies that under these conditions caspase-6 is cleaving itself and is not subject to cleavage and activation by other caspases (Fig 3a, lane 3). In the presence of sox11, cleavage of caspase-6 to remove its prodomain did not occur (Fig 3a, lane 7). While it has been shown in transfected cells that an intact caspase-6 prodomain can prevent all further downstream cleavages [4], we wanted to validate in our system that sox11 is not only capable of inhibiting removal of the prodomain but also inhibits all other cleavage steps. Using an antibody capable of detecting the caspase-6 large subunit, we were able to see sox11 was also capable of inhibiting cleavage of the large subunit (Fig 3a, lower panel). To further validate our findings we performed the same experiment with two other proteins identified from the yeast-two hybrid screen, FEZ1 and COP1 (Fig 3b). In the presence of both FEZ1 and COP1 the band representing caspase-6 following the removal of its prodomain is clearly detected, indicating caspase-6 underwent self-cleavage (Fig 3b). However, in the presence of sox11, caspase-6 self-cleavage is completely blocked (Fig 3b).

To ensure the sox11-mediated affect was not due to a non-specific effect on caspase-6 protein levels, we quantified caspase-6 protein levels in the presence and absence of sox11 via densitometry (Fig 3c). Caspase-6 levels were reduced in the presence of sox11 by 30%. However, caspase-6 activity is decreased by sox11 to control levels (Fig 3d). This therefore indicates that sox11 specifically reduces caspase-6 activity.

### Sox11 Impedes Other Effector Caspases but Not Initiator Caspases

**Fig 4:**
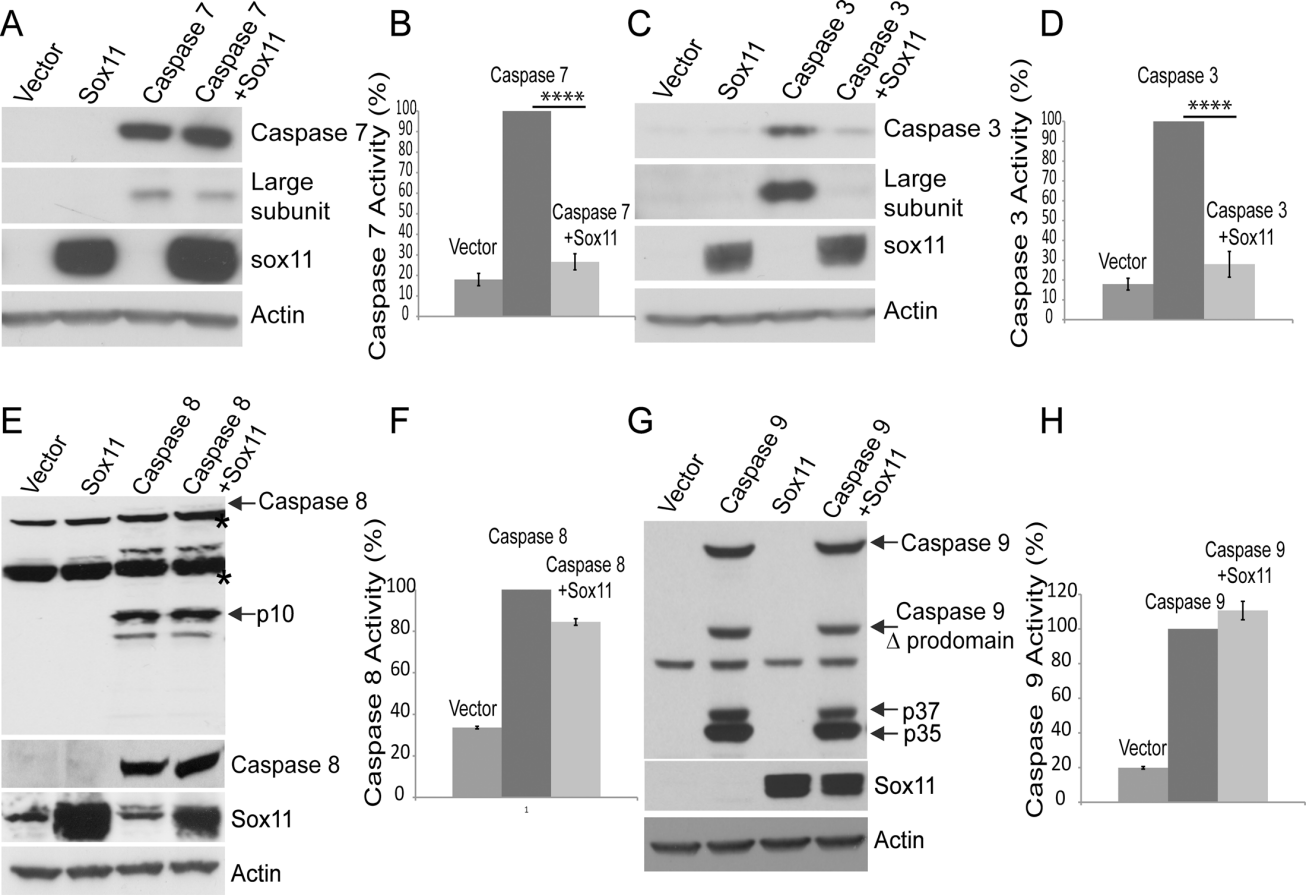
Sox11 reduces caspase-3 and -7 activity but not caspase-8 or -9 activity. (A and C) Top panels show caspase-7 and caspase-3 expression levels with and without sox11 co-expressed. Sox11 co-expression with caspase-3 appears to affect its levels more than caspase-7. The second panel shows the large subunit fragments cleaved from caspase-7 and caspase-3. Both caspase-3 and 7 self- cleavage are reduced. The bottom two panels show sox11 was successfully co-expressed and actin levels indicate protein loading was equal. (B and D) Caspase-3/7 substrate luminescent based assay showed sox11 to reduce both caspase-3 and 7 activities to almost control levels. One-way anova, Sidak’s multiple comparsion test, ****p<0.0001 (E and G) Sox11 co-expression with either caspase-8 or 9 does not affect their steady state levels or proteolysis. (F and H) Caspase-8 and 9 activity assays showed no significant effect of sox11 (n = 3, one-way anova, Sidak’s multiple comparsion test).

Since caspase-6 is an effector caspase, sox11 might also be capable of inhibiting other effector caspases. While co-expression of sox11 with caspase-7 did reduce the amount of its cleaved large subunit, it did not completely block this cleavage event (Fig 4a). Using a substrate based luciferase assay, we were also able to further demonstrate the inhibitory effect of sox11 on caspase-7 activity (Fig 4b). Interestingly, co-expression of sox11 with caspase-3 had a significant impact on both its steady-state levels and its cleavage into large subunit. Sox11 dramatically reduced caspase-3 levels (Fig 4c, **top panel**) and subsequently the levels of its large subunit were also decreased (Fig 4c, **second panel**). We also showed using a substrate based luciferase assay that sox11 was capable of reducing caspase-3 activity to control levels (Fig 4d). The sox11 mediated decrease of caspase-6 activity is not specific to caspase-6, since it also capable of dramatically lowering caspase-7 or -3 activity. Its ability to block caspase-3 self-cleavage may be more likely related to its effect over caspase-3 zymogen levels. Furthermore, considering sox11’s influence over effector caspase activity, we sought to determine if it was also capable of inhibiting initiator caspases. Initiator caspases have a larger prodomain than effector caspases, and are usually activated via clustering and subsequent self-cleavage. Initiator caspases are believed to operate upstream of effector caspases, i.e. initiator caspases first become active and cleave downstream effector caspases, which in turn cleave their appropriate substrate. We next co-expressed sox11 with either caspase-8 or caspase-9 and examined their self-cleavage thereafter. Co-expression of sox11 with either caspase-8 or caspase-9 did not appear to block their capability of self-cleavage (Fig 4e and 4g). Co-expression of sox11 with either caspase-8 or 9 did not affect their zymogen steady state levels or cleavage products (Fig 4e and 4g). We also tested whether sox11 was able to reduce caspase-8 and -9 activity. Using a luciferase based substrate assay, we were able to demonstrate that sox11 had no effect on either caspase-8 or 9 activity (Fig 4f and 4h). Hence, sox11 mediated reduction of caspase activity appears specific to effector caspases.

### Structural Alterations within Sox11 Affect Caspase-6 Activity and Its Interaction with Caspase-6

**Fig 5:**
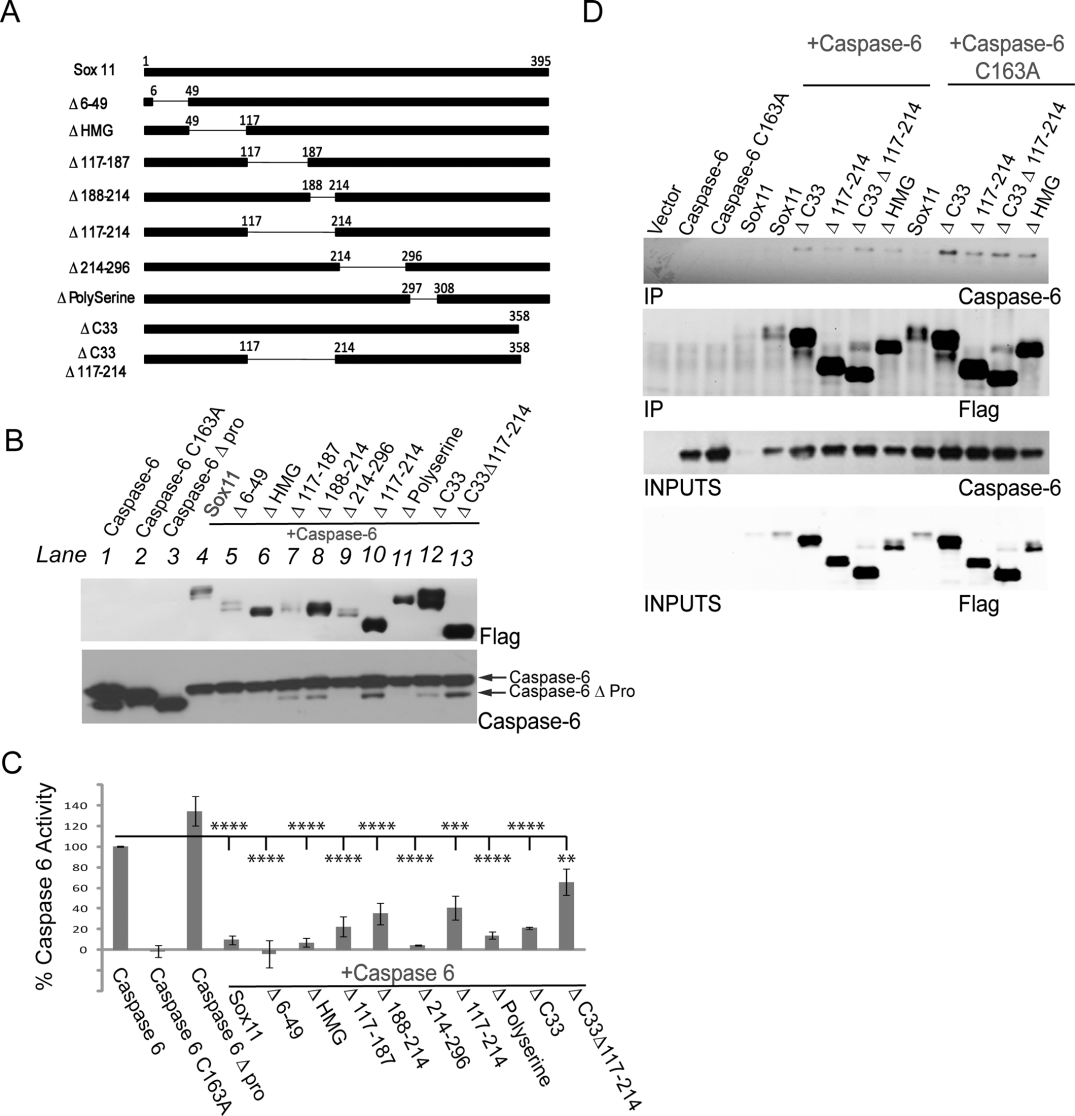
Regions within sox11 contributing to caspase-6 activity decreases include amino acids 117–214, as well as the c-terminal transactivation domain. (A) Schematic diagram depicting sox11 deletion mutants. (B) Caspase-6 substrate assay show all deletions within sox11 caused a significant reduction in caspase-6 activity. Although sox11 mutants Δ117–187, Δ188–214, Δ117–214, ΔC33, and ΔC33 Δ117–214 appeared to cause an increased trend in caspase-6 activity, no significant increase was found when compared to wildtype sox11 (n = 3, one-way anova, Sidak’s multiple comparsion test Sox11 mutants containing deletions of amino acids 6–49, 214–296, or deletion of either the HMG domain or polyserine region retained their capacity to retard caspase-6 activity(C) Western bolt analysis of sox11 mutants (upper panel) show a large difference in expression patterns. Western blots of caspase-6 proteolysis revealed that the sox11 mutants which did not reduce caspase-6 activity to control levels were also incapable of preventing caspase-6 cleavage (lanes 7, 8, 10, 12, 13). (D) Sox11 and mutants were immunoprecipitated from lysates using anti-flag antibody. Caspase-6 was detected using anti-caspase-6 raised against the N-terminus of caspase-6.

The structure of sox11 includes a high mobility (HMG) DNA binding domain near the N-terminus and a transactivational region located at the C-terminus. Additionally, a centrally located acidic region can act as autoinhibitory domain, and prevent non-specific DNA binding [36]. Sox11 deletion mutants were made to determine if any of these or other regions mediated caspase-6 inhibition (Fig 5a). Deletions within sox11 appeared to affect sox11 expression levels. An increase in sox11 steady state levels was observed with deletions of the DNA binding domain (ΔHMG), the central regulatory domain (Δ188–214), and the transactivational domain (ΔC33) and decreases were caused by deletions of regions outside of the known regulatory domains i.e. Δ6–49, Δ117–187, and Δ214–296 (Fig 5b, **top panel**). Interestingly, while sox11 co-expression reduces caspase-6 protein levels, its expression level does not directly correlate with caspase-6 expression levels and prodomain cleavage (Fig 5b, **lower panel**). Note, a decrease in sox11 Δ6–49 expression still holds its negative influence over caspase-6 prodomain cleavage (Fig 5b, **lane 5**). While Δ117–187 (lane7) whose expression is also less than wildtype sox11 (lane 4) partially loses its inhibitory influence over caspase-6 prodomain cleavage. Furthermore, a large increase in sox11 expression caused by deletions of amino acids 188–214, lane 8 and 10 and 358–395 (ΔC33), lane 12 and 13 caused a partial increase of caspase-6 levels. The effects of sox11 mutants on caspase-6 activity were also reflected in the caspase-6 activity assay (Fig 5c).

Since an interaction between sox11 and full length caspase-6 was difficult to detect, perhaps due to rapid turnover, we sought to repeat this interaction study using the sox11 deletions mutants which demonstrated an increase in protein expression. Interestingly, an interaction between full length caspase-6 and its constitutively inactive counterpart caspase-6 C163A was readily detectable with Sox11 deletion mutants ΔC33, Δ117–214, ΔC33Δ117–214, and ΔHMG (Fig 5d). This interaction did appear to be slightly stronger with caspase-6 C163A.

### Sox11 Nuclear Localization Facilitates Its Effect on Caspase-6 Activity

**Fig 6:**
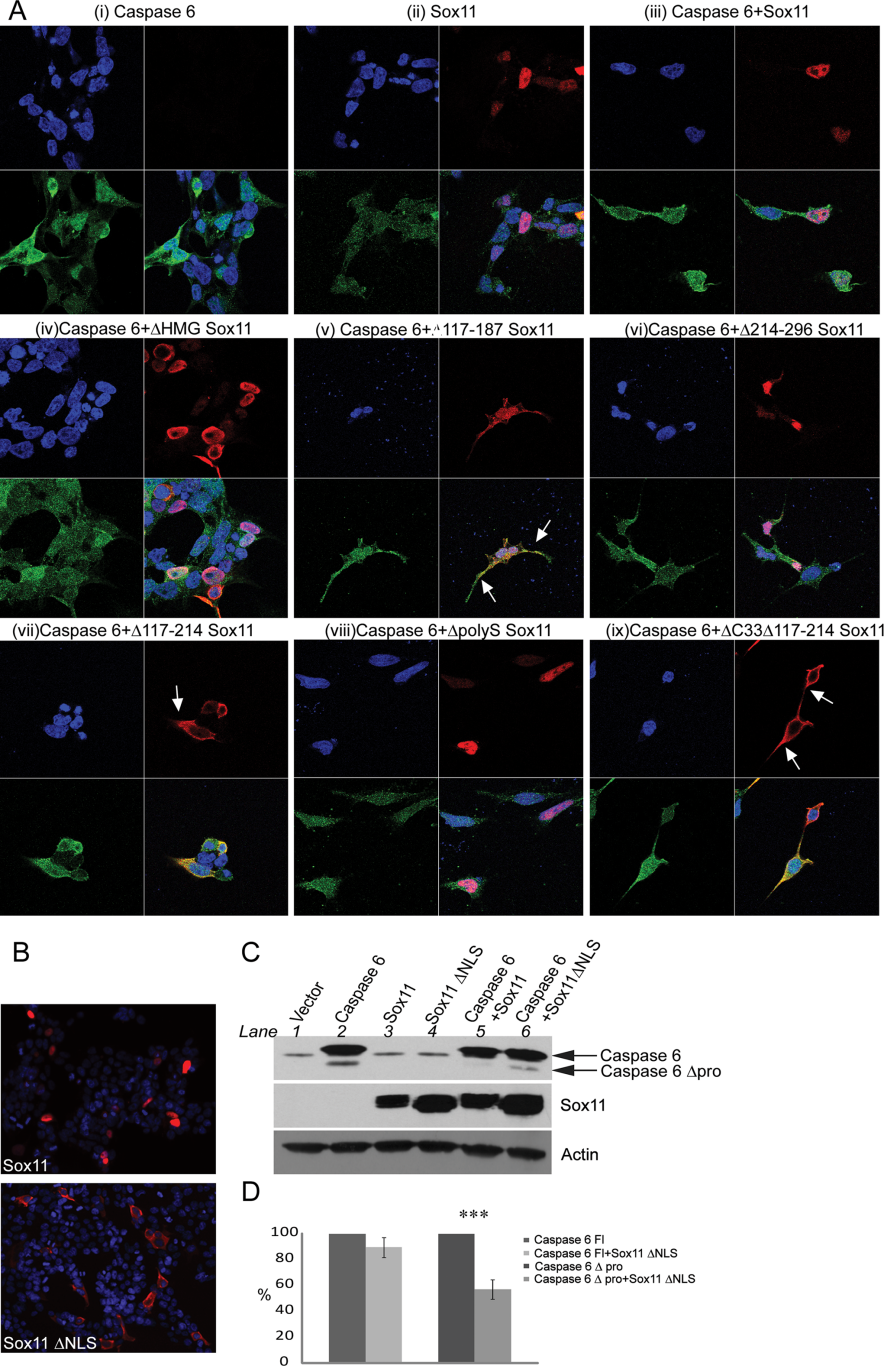
Nuclear localization of sox11 contributes to the loss of caspase-6 activity. (A i-ix) Immunofluorescent images of caspase-6 and sox11, as well as caspase-6 and sox11 deletion mutants captured using a confocal microscope. Several sox11 mutants lose their nuclear localization i.e. (v), (vii), and (ix). Neither sox11 nor sox11 mutants altered the distribution of caspase-6 within the cell. (B) Microscopy images taken to compare the cellular distribution of sox11 and sox11 with the deletion of a putative NLS. Sox11 ΔNLS loses its nuclear localization and becomes more cytoplasmic (lower panel). (C) Western blot comparing caspase-6 proteolysis in the presence of sox11 versus sox11 ΔNLS. Sox11 ΔNLS is no longer capable of completely blocking caspase-6 cleavage (compare lanes 5 and 6). (D) Quantification of caspase-6 FL (full length, zymogen) levels vs caspase-6 post prodomain cleavage with and without sox11 ΔNLS from (C). No significant change was seen in caspase-6 full length levels in the presence of sox11 ΔNLS. However, caspase-6 prodomain levels were increased 56% in the presence of sox11 ΔNLS.

In order to investigate whether the subcellular localization of caspase-6 was altered by sox11 or sox11 mutants, we performed confocal microscopy studies. Caspase-6 has a diffuse distribution throughout the cell while sox11 is located only in the nucleus (Fig 6a(i) and 6a(ii), respectively). Both caspase-6 and sox11 colocalize inside the nucleus (Fig 6a(iii)). Interestingly, while neither sox11 nor its mutants affected caspase-6 distribution, the subcellular localization of several sox11 mutants was altered. Notably, sox11 mutants Δ117–187, Δ117–214, and Δ33Δ117–187 which were less capable of blocking caspase-6 activity became less nuclear and more cytoplasmic (Fig 5a (v), (vii), (ix)). Hence, nuclear localization of Sox11 is likely very important for its ability to inhibit caspase-6 cleavage.

A consensus sequence for a monopartitie NLS exists just outside the HMG domain between amino acids 122–125 (KKPK). To determine if this was indeed an NLS, these four amino acids were deleted. This sequence is important for Sox11 nuclear localization since its deletion resulted in a more cytoplasmic distribution (Fig 6b, sox11 ΔNLS). Furthermore, co-expression of sox11 without an NLS with caspase-6 allowed for caspase-6 self-cleavage to return but to a lesser extent than caspase-6 expressed alone (Fig 6c, compare lane 2 and 6). In order to quantify this effect, caspase-6 zymogen levels were set at 100% and co-transfection of sox11 ΔNLS with caspase-6 was expressed as a percentage of this (Fig 6d). No significant difference in steady state levels was detected between transfection of caspase-6 alone versus co-transfection with sox11 ΔNLS. The same method was used to calculate the percentage difference between the amount of cleavage taken place in the presence of caspase-6 alone versus its co-transfection with sox11 ΔNLS (Fig 6d). Caspase-6 Δprodomain levels were reduced by approximately 50% in the presence of sox11 ΔNLS. While sox11 nuclear localization is important for sox11 mediated caspase-6 inhibition, it is not solely responsible since caspase-6 self-cleavage does not return to the same levels as when expressed alone.

### Sox11 Can Inhibit Toxicity in Primary Neurons and Other Sox Proteins Can Affect Caspase-6 Activity

**Fig 7:**
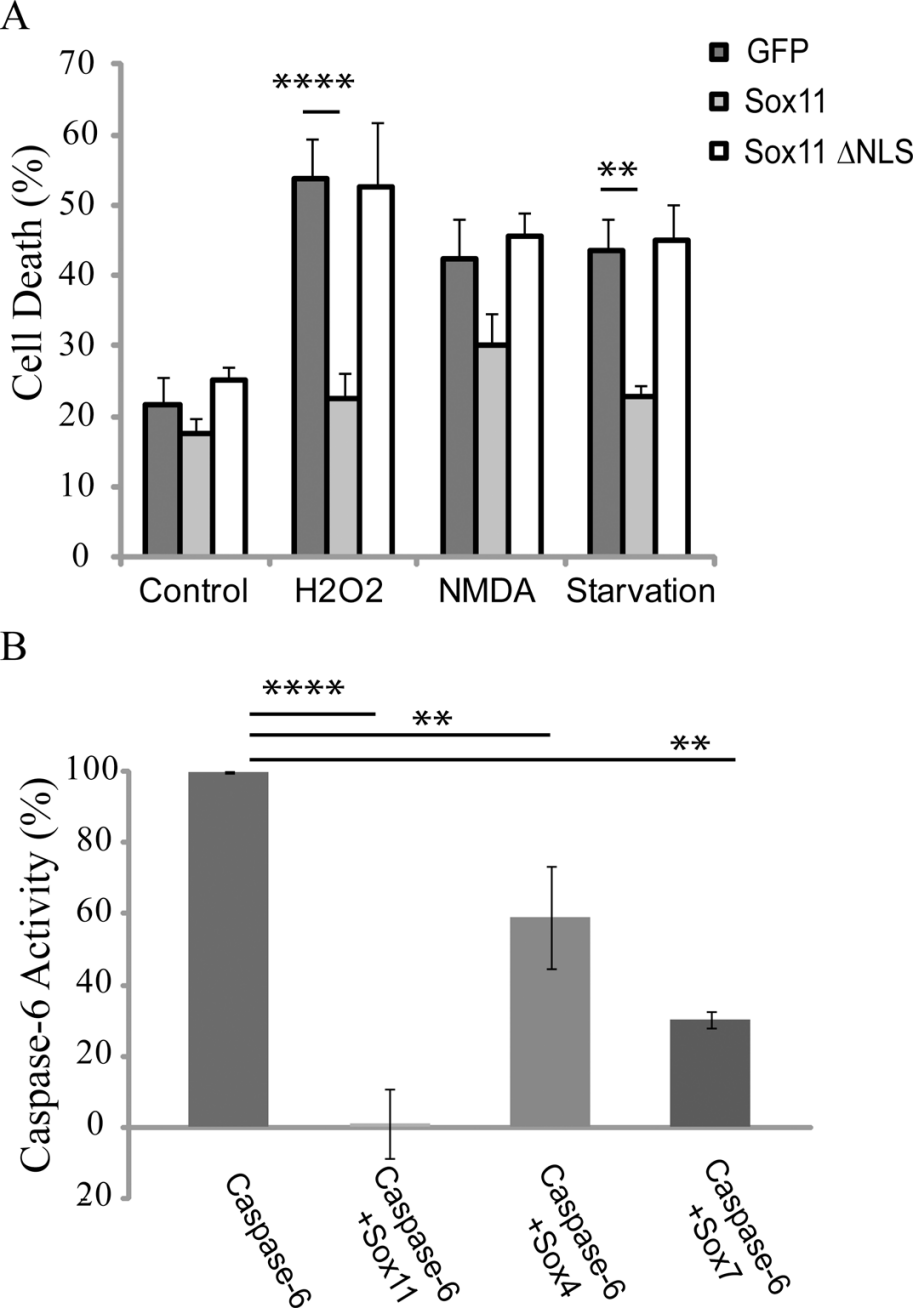
Sox11 is protective against apoptotic insult and other sox proteins can also reduce caspase-6 activity. (A) Mouse cortical primary neurons were transfected with either sox11 or sox11 ΔNLS. 24 hours post-transfection, neurons were either treated with toxic agents or subjected to serum starvation for a further 24 hours. Neurons were fixed and stained and assessed for cell death via nuclear condensation (n = 8, one-way anova, Sidak’s multiple comparison test ****p<0.0001, **p<0.01). (B) Caspase-6 activity was measured using a luciferase-based substrate assay from cells transfected with indicated plasmids. Sox11 reduced caspase-6 activity to control levels. Sox4 and sox7 partially reduced caspase-6 activation (n = 2, one-way anova, Sidak’s multiple comparison test ****p<0.0001, **p<0.01).

Activation of effector caspases is a terminal event during end stage apoptosis. To investigate whether sox11 could prevent cell toxicity, cortical mouse primary neurons were prepared and transfected with sox11 as well as sox11 ΔNLS and treated with several toxic agents (Fig 7a). Cell death was assayed using a previously described nuclear condensation assay [37]. Interestingly, transfection of sox11 was capable of preventing toxicity in primary neurons which were treated with either H_2_O_2_ or subjected to serum starvation. However, sox11 was incapable of preventing cell death induced using NMDA. Furthermore, sox11 ΔNLS which was ineffective at decreasing caspase-6 activity was likewise ineffective at preventing toxicity in neurons treated with H_2_O_2_ or serum starved (Fig 7a). Sox11 may only be able to prevent toxicity induced cell death via its capacity to inhibit effector caspases.

The ability of sox11 to reduce caspase-6 activity might extend to other sox family members. For this reason caspase-6 was co-expressed with another SoxC family member, sox4 or with sox7, a member of the SoxF family. With exception to the DNA binding domain, close homology in protein structure and amino acid sequence is only found within the sox subfamily [38]. Both sox4 and sox7 were capable of partially lowering caspase-6 activity. Sox7, which is part of a different subgroup was better able to reduce caspase-6 activity than sox4 (Fig 7b). Thus, sox11’s capacity for decreasing caspase-6 activity may be shared by other sox proteins.

## Discussion

Our yeast two-hybrid screen using catalytically inactive caspase-6 was successful in identifying sox11 as a possible binding partner. Sox11 or SRY (sex-determining region Y)-box 11 is a transcription factor and has important roles in embryonic development and cell fate [30,31]. This study was tailored for the identification of regulatory interactions rather than additional substrates. Since over-expression of caspase-6 causes self activation, we used caspase-6 C163A as bait. Supporting rationale for choosing caspase-6 C163A for bait include a previous yeast-two-hybrid screen using constitutively active caspase-7, which was successful in identifying novel substrates [39]. Hence, using a catalytically inactive caspase for bait may be better suited to pick out regulatory binding proteins. Furthermore, it has been shown in the case of kinases that some inhibitors bind preferentially to the inactive conformation of the enzyme [40].

Sox11 was identified as a candidate binding protein and appears to have a strong ability for lowering caspase-6 activity. The exact mechanism responsible for this inhibition is not straight forward and may involve more than one mode of action elicited by sox11. From these studies, it can be appreciated that sox11 causes a reduction in caspase-6 protein levels but not to the extent which can account for a complete reduction in enzyme activity. Sox11 also appears capable of preventing caspase-6 autocleavage, an event which would prevent caspase-6 activation. It is unlikely under these conditions that caspase-6 is being cleaved by another endogenous effector caspase since sox11 is capable of blocking their activity too. It is also unlikely that caspase-6 is being cleaved by an initiator caspase since no agents or conditions were used to activate them. Caspase-6 must dimerize in order to be able to cleave itself. Sox11 may act by preventing dimerization of caspase-6.

Interestingly, sox11 had no effect on caspase-7 protein levels but almost completely decreased its activity. Sox11 did however have a significant influence over caspase-3 protein levels. If decreasing effector caspase protein levels is part of its mode of action, then there might be a hierarchy of targets with caspase-3 being first, caspase-6 in the middle, and caspase-7 last. Moreover, the specificity of sox11 mediated effect towards effector caspases and not initiator caspases is intriguing. By contrast members of the IAP family inhibit both effector and initiator caspases. Caspases have functions outside of cell death and perhaps sox11’s unique capability to block effector caspase activity allows initiator caspases to carry out other functions without stimulating cell death [3]. This capability might also be relevant during development for which sox11 has a very important role [30,31]. Sox11’s ability to lower caspases-6, -3, and -7 activities might also be relevant to cancer, since carcinogenesis involves an escape from normal apoptosis. In addition, sox11 levels have been found to be elevated in certain cancers in particular, mantle cell lymphoma, medullablastomas, Burkitt lymphoma, and immature lymphocytic neoplasm [41,42].

The co-immunoprecipitation of caspase-6 and sox11 in cells was difficult. Initial attempts to co-immunoprecipitate full length caspase-6 failed. This led us to posit that the interaction between sox11 and caspase-6 may be transient. Therefore, to increase the likelihood of detecting a positive interaction between these two proteins, we used the caspase-6 large and small subunits tagged with GFP to increase their stability. Interestingly, we only detected a positive interaction with the large subunit of caspase-6 C163A. Constitively inactive caspase-6 may have a longer half-life than its wild type counterpart and therefore may have aided in the detection of this interaction. Indeed the turnover of sox11 might also factor in to the detection efficiency of this interaction. Deletions within key domains of sox11 allowed us to more readily detect an interaction with both wild type and inactive caspase-6. The interaction between sox11 and full length caspase-6 may have become more stable due to the reduction in sox11 turnover caused by the mutation. It is likely the interaction between sox11 and caspase-6 is transient and can only be clearly demonstrated using mutations which allow sox11 to evade degradation. Interestingly, while sox11 ΔHMG can still negatively impact caspase-6 activation, the interaction between it and caspase-6 is clearly detected.

Efforts to address regions within sox11 mediating its effect on caspase-6 were tricky to interpret due to the effect of the deletion on sox11 expression levels. However, increased sox11 expression did not cause an artificial decrease in caspase-6 proteins levels and likewise lower expression of sox11 did not result in an increase in caspase-6 amounts. Therefore, the effect of sox11 on caspase-6 cannot be explained by its expression levels. Since sox11 is a transcription factor, there is also the possibility it induces the transcription of an additional protein which could mediate inhibition of caspase-6 activity. The regions participating in sox11-mediated inhibition of caspase-6 have previously been identified as areas responsible for its transcriptional and transactivational potential. However, deletion of the sox11 DNA binding domain which should inhibit its capacity as a transcription factor did not cause an increase in caspase-6 activity. Transcription factors and those from the sox family often work together to control cellular events [43,44]. It might also be possible that sox11 is cooperating with another transcription factor to induce the transcription of an intermediate protein.

The importance of sox11 nuclear localization was demonstrated by deleting its NLS. The sox11 mutants which were shown to partially lose their influence over caspase-6 activity displayed an altered cellular expression profile i.e., less nuclear and more cytoplasmic, which further supports the importance of a nuclear sox11. The relevance of sox11 nuclear localization to caspase-6 inhibition might be because caspase-6 is mainly active inside the nucleus [45,46,47]. Hence, if sox11 is not present in the nucleus, caspase-6 can become active. The nuclear location of soxll and its influence over caspase-6 activity might suggest a role for it in preventing caspase-6 mediated cleavage of lamin A/C. Nuclear lamins were the first described substrates for caspase-6 and their cleavage is one of the final stages of apoptosis. Lamin A/C have been shown to be exclusively cleaved by caspase-6 [48]. This inhibitory action mediated by sox11 may be a necessary safety measure in preventing accidental or unwanted cell death.

The effect of sox11 over caspase-6 was not specific to sox11. Sox4 as well as Sox7 also demonstrated reduction in caspase-6 activity. It may be that different sox proteins or different families of sox proteins have the potential to regulate different caspases at different time points. This would place the sox proteins as master regulators of differentiation and cell death. The function of sox11 in the proliferation and survival of osteoblast lineage cells has been demonstrated using siRNA and toxicity assays. The authors showed increased amounts of cleaved caspase-3 in the presence of sox11 siRNA [49]. This further supports the ability of sox11 to lower effector caspase activity and increase cell survival and also implies new functional roles for sox proteins.

Activation of effector caspases is also a key event mediating neuronal death in neurodegenerative disease [12]. The importance of caspase-6 during brain development has been established by its elimination in mice [50,51]. Caspase-6 deficient mice develop a hypoactive phenotype and display learning deficits. Additionally, caspase-6 -/- neurons are protected from axonal degeneration induced by either nerve growth factor deprivation or NMDA mediated excitotoxicity. Interestingly, the regions most affected are areas vulnerable to neurodegenration in several disease states, especially Huntington’s disease, i.e. cortex and striatum [50]. Proteins central to neurodegenerative diseases, such as Htt in HD, as well as amyloid precursor protein (APP), tau, and presenilin in Alzheimer’s disease (AD) are all caspase-6 substrates. Furthermore, active caspase-6 levels are suggested to be elevated in both HD and AD [18,20]. Given the pivotal role played by effector caspases in neurodegeneration and its ability to increase neuronal survival after stress, sox11 could be a potential therapeutic candidate.

To date, no naturally expressed inhibitor for caspase-6 has been described. Structures of different conformations of caspase-6 have recently been solved [52], and suggest the possibility of an allosteric site which can mediate inhibition [53,54,55]. Identification of inhibitory interactions, in combination with structural information, could potentially provide clues to the design of new classes of small molecule inhibitors. While the exact mechanism for by which sox11 reduces caspase-6 activity remains to be determined, sox11 is the first naturally expressed protein capable of dramatically reducing caspase-6 activity and may function as an inhibitor. This finding merits further investigation into the role of Sox family members in caspase regulation and their potential to be classified as novel caspase inhibitors.

## Materials and Methods

### Yeast Two Hybrid Screening

The Matchmaker^™^ Two-Hybrid System 3 and pretransformed human fetal brain library were both purchased from Clontech. Yeast transformations were carried out using the yeastmaker yeast transformation system 2 (Clontech). Prior to library screening, the bait plasmid was tested for autoactivation and toxicity according to manual instructions. Library screening was carried out at the highest stringency possible using three reporters (-Ade/-His/-Leu/-Trp/ X-α-Gal).

### Cell Culture and Transfections

HEK 293FT cells were maintained in Dulbecco’s modified media (Gibco) containing 10% FCS (Quality Biological), 0.5mg/ml G418 sulfate (Cellgro) and 1x penicillin/streptomycin (Invitrogen), in a humidified 5% CO_2_ incubator at 37°C. Cells were transfected when approximately 70% confluent using lipofectamine 2000 (Invitrogen) at a DNA: lipo ratio of 1:2 according to standard protocols. Cells were harvested 24hours post-transfection and lysed on ice using an NP-40 buffer (50 mM Tris, 150 mM NaCl, 1% NP40, 0.02% NaN_3_) supplemented with complete protease inhibitor (Roche). Protein concentration was determined by the BCA method (Pierce)

### cDNA Constructs, Mutagenesis and Cloning

pET23b pro-caspase-6 was obtained from ATCC (ATCC 99626) and cloned into the pCDNA3 expression vector. The catalytic cysteine residue at position 163 was mutated to an alanine using the lightning mutagenesis kit (stratagene). Caspase-6 C163A was cloned into the yeast expression vector pGBKT7. To enable a more clear separation of caspase-6 and its prodomain in western blot analysis, a flag tag was added to the caspase-6 N-terminus and cloned into the expression vector pCI-neo (promega). Using caspase-6 as template, the prodomain was omitted via pcr beginning at the large subunit. Caspase-6 Δprodomain was then cloned into pCI-neo. Caspase-6 subunits were constructed via conventional pcr and cloned in to EGFP N3 (clontech). pET23b Caspase-3 His (addgene 11821), pCDNA3 Flag caspase-7 (addgene 11815), pCDNA3 caspase-8 (addgene 11817), pET23b caspase-9 His (addgene 11829), were all deposited by Guy Salvesen. pET23b Caspase-8 and caspase-9 were used as templates for amplification and were subsequently cloned into the EGFP N3 expression vector. pCMV5-Flag Sox11 (rat) was a kind gift from Angie Rizzino. All sox11 deletion mutants were constructed using conventional pcr strategies. In brief, pCMV5 flag sox11 was used as a template and amplified with the omission of a given region. Both 5’and 3’ primers were created with a HindIII overhang to allow for restriction digest and ligation of the plasmid. Sox11 ΔNLS was constructed using the lightning mutagenesis kit (stratagene). The 6FXO-p89Luc reporter and pCDNA6 V5 HisSox4 and Sox7 were generously provided to us by Veronique Lefebvre. All cloning was performed using infusion pcr cloning system (clontech) according to manufacturer’s instructions.

### RT-PCR

To analyze caspase-6 mRNA levels upon co-expression with sox11, total RNA was extracted using RNeasy^®^ mini kit (Qiagen). Reverse transcription and PCR was carried out on 50ng RNA using OneStep RT-PCR kit (Qiagen). The following primers were used caspase-6 forward 5’-GGACCACAGGAGGAGAGGAATTGC -3’, reverse 5’-GCACATCGTGCTGGTTTCCCCGAC -3’; GAPDH forward 5’-CTACTGGCGCTGCCAAGGCTGTGGG -3’, reverse 5’-CCTTGGAGGCCATGTGGGCCATGAG -3’. After an initial 15 min activation step, twenty five PCR cycles were carried out. PCR conditions were 94°C for 1min, 59°C for 1min and 72°C for 1min.

### Western Blotting and Caspase-6 Substrate Assay

Equal amounts of whole cell lysate were separated on a 4–12% Bis-Tris polyacrylamide gel (Novex). Proteins were transferred onto nitrocellulose membrane (Bio-Rad) and immunoblotting was carried out using the following antibodies: all caspase antibodies were purchased from cell signalling and used at a 1/1000 dilution, caspase-6 (9762), caspase-8 (4790), cleaved caspase-7 (9491), caspase-9 (9505), cleaved caspase-3 (9661). Both caspase-8 and caspase-9 could also be detected using anti-GFP (Roche, 1/10000). Immunoprecipitation experiments were carried out using Dynabeads (invitrogen) according to manufacturer’s instructions. Caspase-6 subunits were pulled down using anti-GFP antibody at a 1/1000 dilution. Sox11 was detected using anti-flag antibody (sigma, 1/10000). Antibody reactivity was detected using enhanced chemiluminescence (GE healthcare). The coimmunoprecipitation of sox11 and its deletion mutants was performed using Dynabeads and anti-flag antibody. Full length caspase-6 was detected using anti-caspase-6 antibody (sigma, 1/1000). Caspase-6 activity was determined from whole cell lysates 24hours post-transfection using a luciferase assay reporter system (caspase-Glo^®^ 6, Promega) according to manufacturer’s instructions. Briefly, the assay relies on a proluminescent caspase substrate, which contains the tetrapeptide cleavage sequence VEID for caspase-6 and DEVD for caspases-3 and -7. The tetrapeptide sequence is cleaved to release the luciferase substrate aminoluciferin, used in the production of light. The amount of light measured will thus correspond the amount of caspase activity.

Immunofluoresence of caspase-6 and sox11 was performed using standard protocols. Briefly, cells were fixed for 5 min at room temperature with 4% paraformaldehyde in PBS. Cells were washed with PBS and cell membranes were permeabilised using 0.1% Triton X-100 in PBS (PTx). Non-specific binding was blocked using 4% goat serum for 1 h at room temperature. Primary and secondary antibodies were diluted in blocking buffer and incubation periods were conducted for 1 h at room temperature, followed by 3 washes with PTx buffer. Caspase-6 was detected using anti-caspase-6 (cell signalling, 9762) at 1/100. Sox11 was imagined using anti-flag (sigma) at 1/1000. FITC and Cy3-labeled secondary antibodies were used subsequently. Microscopy was performed using an LSM 510 Meta laser scanning confocal microscope equipped with a 63X objective. Micrographs were arranged and exported using LSM Image Browser.

### Nuclear Condensation Assay

Primary cortical neurons were isolated from embryonic day 17 mice. Neurons were co-transfected with GFP and either sox11 or sox11ΔNLS at DIV7 using Lipofectamine 2000 according to the manufacturer’s protocol. After 24h, the cells were treated with H_2_O_2_, glutamate, or serum starved for a further 24h. Cells were then fixed for 30 min with 4% paraformaldehyde in PBS. After 3 washes with PBS, cells were treated with 0.8 μg/mL of bisbenzimide (Hoechst 33342, Sigma). Cells were automatically analyzed using an inverted fluorescence microscope (Axiovert 200, Zeiss) and images were digitized from 144 independent fields per well. Transfected cells were visualized via GFP fluorescence and quantified for cell survival using the Volocity software (Perkin Elmer) by automated measurement of the average intensity of Hoechst stained nuclei of transfected cells. Cells were considered viable when their intensity was lower than 200% of the control intensity.

## Abbreviations

IAP: inhibitor of apoptosis
NLS: nuclear localization signal
HD: Huntington’s disease
AD: Alzheimer’s disease
